# Tuberculous Toxaemia and Temperament

**Published:** 1913-02

**Authors:** 


### ABSTRACTS

Tuberculous Toxaemia and Temperament.—The editor of the New York *Medical Journal,* December 7, 1912, mentions the following geniuses who have succumbed to tuberculosis, strongly implying that their peculiar temperament, if not artistic ability, was due to the disease: Marie Bashkirtseff, her platonic affinity, Bastien Le Page, who painted the Joan of Arc, Chopin, Robert Louis Stevenson, Rachel, Stephen Crane, Schiller, Laurence Sterne, John Keats, Nevin, von Weber, John Sterling, Timrod, Artemas Ward, Henry Kirk White, Thoreau, Spinoza, Merimee, Symonds, Kingley, Southey, Shelley, Pollock, Pope, Hood, de Quincy, de Balzac, Hawthorne, Burns, Poe, Coppee, John Stuart Mill, Richelieu, Descartes, Cowper, Moliere, Botticelli's model, Simonetta Catanea, and the model who sat for the Blessed Damozel of Rosetti.

There certainly is an analogy in the literary conceptions, music and pictorial art of the respective geniuses and it is, perhaps, not far fetched, to find an analogy, though difficult to express, among the artistic peculiarities in these respective fields.

